# Supplementary material for: Short Term Caloric Restriction and Biofeedback Enhance Psychological Wellbeing and Reduce Overweight in Healthy Women
Source: J Pers Med. 2021 Oct 26;11(11):1096. doi: 10.3390/jpm11111096 (PMC8623687; doi:10.3390/jpm11111096)
Supplement: Supplementary file 1 [file jpm-11-01096-s001.zip › jpm-1414565-supplementary.pdf]

Parameters<br>Total (n, Mean ± SD)	Group	Visit 1<br>Mean ± SD	n	Visit 2<br>Mean ± SD	n
**Serum Parameters**					
**Adiponectin**<br>V1 41, 11.7 ± 4.1<br>V2 41, 11.1 ± 4.1	CR<br>CR + CPI	12.2 + 4.8<br>11 + 3.1	21<br>20	11.9 + 5.3<br>10.4 + 2.4	20<br>21
**Leptin**<br>V1 41, 15.9 ± 10.7<br>V2 41, 9.5 ± 7.8	CR<br>CR + CPI	12 + 8.4<br>20 + 11.5	21<br>20	6.4 + 5.4<br>12.6 + 8.6	20<br>21
**Secretagogin**<br>V1 41, 151 ± 157.5<br>V2 41, 128.1 ± 133.1	CR<br>CR + CPI	174 + 175<br>126.9 + 137.1	21<br>20	143.4 + 144.4<br>113.5 + 123	20<br>21
**Resistin**<br>V1 41, 7.7 ± 3.4<br>V2 41, 7 ± 2.8	CR<br>CR + CPI	7.1 + 3.7<br>8.3 + 3	21<br>20	6.6 + 2.8<br>7.4 + 2.8	20<br>21
**HDL Cholesterol**<br>V1 43, 1.8 ± 0.4<br>V2 42, 1.6 ± 0.4	CR<br>CR + CPI	1.9 + 0.4<br>1.6 + 0.3	22<br>21	1.7 + 0.4<br>1.4 + 0.3	21<br>21
**Total Cholesterol**<br>V1 43, 5.6 ± 1.2<br>V2 42, 5.3 ± 1.4	CR<br>CR + CPI	5.7 + 1.1<br>5.4 + 1.2	22<br>21	5.4 + 1.5<br>5.2 + 1.3	21<br>21
**proBNP**<br>V1 43, 104.9 ± 142.2<br>V2 41, 82 ± 100.2	CR<br>CR + CPI	141.2 + 182.9<br>66.9 + 66.1	22<br>21	107.9 + 122.3<br>57.4 + 67.7	20<br>21
**AP**<br>V1 43, 67.3 ± 18.8<br>V2 41, 61.6 ± 17.7	CR<br>CR + CPI	65.8 + 17<br>68.9 + 20.8	22<br>21	60.2 + 16.8<br>63 + 18.8	20<br>21
**GGT**<br>V1 43, 21.2 ± 19.5<br>V2 41, 17.3 ± 14.1	CR<br>CR + CPI	17.4 + 8.1<br>25.2 + 26.7	22<br>21	14.3 + 6.6<br>20.1 + 18.5	20<br>21
**BDNF**<br>V1 41, 17977.9 ± 12006.9<br>V2 41, 17613.3 ± 12278.1	CR<br>CR + CPI	19898 + 12930<br>15960 + 10915	21<br>20	21939 + 14082<br>13492 + 8749	20<br>21
**IL-6**<br>V1 41, 2.9 ± 2<br>V2 40, 2.8 ± 1.5	CR<br>CR + CPI	1.9 + 0.6<br>3.8 + 2.5	20<br>21	2.1 + 1<br>3.4 + 1.7	19<br>21
**CRP**<br>V1 43, 0.4 ± 0.4<br>V2 42, 0.4 ± 0.3	CR<br>CR + CPI	0.23 + 0.23<br>0.5 + 0.5	22<br>21	0.29 + 0.3<br>0.45 + 0.36	21<br>21
**Cortisol (Serum)**<br>V1 43, 15.7 ± 4.8<br>V2 41, 15.7 ± 4.8	CR<br>CR + CPI	16.3 + 4.7<br>15.1 + 4.9	22<br>21	15.2 + 4.5<br>16.1 + 5.1	20<br>21
**Cortisol (Sputum)**<br>V1 43, 0.5 ± 0.3<br>V2 39, 0.4 ± 0.2	CR<br>CR + CPI	0.51 + 0.28<br>0.49 + 0.3	22<br>21	0.44 + 0.25<br>0.48 + 0.2	20<br>19
**Physical Activity**	CR<br>CR + CPI	50.9 + 23.4<br>36.3 + 12	16<br>16	/<br>/	/<br>/

Parameters<br>Total (n, Mean ± SD)	Group	Visit 1<br>Mean ± SD	n	Visit 2<br>Mean ± SD	n
**Anthropometric Parameters**					
**BMI**<br>V1 43, 27.6 ± 5.7<br>V2 43, 26.7 ± 5.6	CR<br>CR + CPI	26.2 ± 4.2<br>29 ± 6.8	22<br>21	25.5 ± 4.2<br>27.9 ± 6.5	22<br>21
**Waist Circumference**<br>V1 43, 97.9 ± 14.2<br>V2 40, 94.7 ± 12.7	CR<br>CR + CPI	97.3 ± 12.3<br>98.6 ± 16.2	22<br>21	93.3 ± 9<br>96.1 ± 16.1	20<br>20
**WHtR**<br>V1 43, 1.7 ± 0.2<br>V2 42, 1.8 ± 0.2	CR<br>CR + CPI	1.7 ± 0.2<br>1.7 ± 0.3	22<br>21	1.8 ± 0.2<br>1.8 ± 0.3	21<br>21
**Bodyfat**<br>V1 31, 27.7 ± 13.1<br>V2 23, 26.6 v 12.2	CR<br>CR + CPI	24.1 ± 10.6<br>31 ± 15.1	19<br>12	23.5 ± 11.8<br>26.5 ± 11.2	14<br>9
**Lean Mass**<br>V1 31, 47.7 ± 5.4<br>V2 24, 47 ± 4.7	CR<br>CR + CPI	47 ± 4.4<br>48.6 ± 6.9	19<br>12	47 ± 4.17<br>46.8 ± 5.6	14<br>10
**FLI**<br>V1 43, 41.2 ± 28.9<br>V2 41, 35.3 ± 26.8	CR<br>CR + CPI	32.8 ± 21.8<br>50 ± 33.2	22<br>21	27.3 ± 21.5<br>42.9 ± 29.5	20<br>21
**Hormones**					
**Estradiol**<br>V1 43, 49.6 ± 70.1<br>V2 41, 45.8 ± 83.3	CR<br>CR + CPI	54.2 + 88.4<br>44.7 + 45.4	22<br>21	33.8 + 50.5<br>57.3 + 105.6	20<br>21
**Testosterone**<br>V1 43, 0.22 ± 0.2<br>V2 41, 0.24 ± 0.2	CR<br>CR + CPI	0.17 + 0.1<br>0.27 + 0.2	22<br>21	0.18 + 0.1<br>0.3 + 0.2	20<br>21
**FSH**<br>V1 43, 51.3 ± 36.4<br>V2 41, 60.4 ± 43.7	CR<br>CR + CPI	51.9 + 33<br>50.7 + 40.4	22<br>21	58.8 + 40.7<br>61.9 + 47.3	20<br>21
**LH**<br>V1 43, 29.6 ± 19.3<br>V2 41, 31 ± 19.8	CR<br>CR + CPI	30 + 17.3<br>29.3 + 21.7	22<br>21	28.7 + 17.5<br>33.2 + 22	20<br>21
**Biofeedback Parameters**					
**HRV Baseline**<br>V1 42, 13.3 ± 12.7<br>V2 38, 11.9 ± 8.5	CR<br>CR + CPI	17.1 + 6.7<br>9.5 + 4.9	21<br>21	13.9 + 10.4<br>10.1 + 6.1	18<br>20
**HRV Stress**<br>V1 42, 16.8 ± 12.9<br>V2 38, 14.3 ± 9.4	CR<br>CR + CPI	21.9 + 16.1<br>11.6 + 4.8	21<br>21	18.5 + 11.4<br>10.6 + 5	18<br>20
**Systolic Blood Pressure**<br>V1 42, 126.2 ± 18.8<br>V2 43, 119 ± 15.8	CR<br>CR + CPI	126.2 + 20.2<br>126.3 + 17.8	21<br>21	120.1 + 11.5<br>117.7 + 19.6	22<br>21
**Diastolic Blood Pressure**<br>V1 42, 84 ± 11.4<br>V2 43, 81.4 ± 11.6	CR<br>CR + CPI	83.5 + 11.1<br>84.4 + 11.9	21<br>21	81.7 + 8.4<br>81 + 14.5	22<br>21

**Psychometric Parameters**					
**PSS**<br>V1 33, 15.3 ± 5.1<br>V2 33, 12 ± 5.8	CR<br>CR + CPI	13.8 + 5.2<br>16.6 + 4.8	16<br>17	12.1 + 5.3<br>12 + 6.4	16<br>17
**Stress (Family Life)**<br>V1 39, 45.7 ± 30.5<br>V2 36, 47.7 ± 27.8	CR<br>CR + CPI	40.9 + 31.5<br>50.8 + 29.4	20<br>19	45.2 + 28.3<br>50.6 + 27.8	19<br>17
**Stress (Work Life)**<br>V1 38, 50 ± 35<br>V2 35, 50.3 ± 28.1	CR<br>CR + CPI	50.2 + 35.3<br>49.7 + 35.6	20<br>18	49.1 + 27.5<br>51.6 + 29.4	19<br>17
**Stress (Self-Oriented)**<br>V1 39, 56 ± 24.6<br>V2 36, 47.3 ± 27.3	CR<br>CR + CPI	47.5 + 28<br>64.9 + 16.9	20<br>19	43.2 + 29.2<br>51.9 + 25.2	19<br>17
**GSI (BSI)**<br>V1 43, 56.4 ± 10.3<br>V2 43, 46.8 ± 10.8	CR<br>CR + CPI	56.2 + 11.4<br>56.7 + 9.1	22<br>21	45 + 11.5<br>48.8 + 9.8	22<br>21
**Anxiety (BSI)**<br>V1 43, 51.8 ± 7.9<br>V2 43, 49 ± 6	CR<br>CR + CPI	55.2 + 11.9<br>55.5 + 8.9	18<br>19	44.1 + 8.5<br>49.5 +8.9	15<br>17
**Depression (BSI)**<br>V1 43, 54.7 ± 9.3<br>V2 43, 48.4 ± 7.7	CR<br>CR + CPI	55 + 10.1<br>54.3 + 8.7	18<br>19	47.5 + 7.9<br>49.3 +7.5	15<br>17
**Somatization (BSI)**<br>V1 43, 56.1 ± 10.2<br>V2 43, 52 ± 10.5	CR<br>CR + CPI	54.4 + 10.5<br>50.1 + 10.3	18<br>19	58 + 9.7<br>53.9 + 10.5	15<br>17
**Paranoia (BSI)**<br>V1 43, 54.4 ± 8.8<br>V2 43, 48.5 ± 7	CR<br>CR + CPI	55.1 + 8.9<br>53.7 + 8.8	18<br>19	47.5 + 6.8<br>49.5 + 7.2	15<br>17
**Psychoticism (BSI)**<br>V1 43, 53.4 ± 7.4<br>V2 43, 48.4 ± 6	CR<br>CR + CPI	54 + 7.6<br>52.7 + 7.3	18<br>19	48 + 6.4<br>48.8 + 5.8	15<br>17
**Social Insecurity (BSI)**<br>V1 43, 54.9 ± 9.5<br>V2 43, 47.4 ± 8.4	CR<br>CR + CPI	54.3 + 10.5<br>55.5 + 8.6	18<br>19	47.6 + 9.9<br>47.2 + 6.7	15<br>17
**Aggression (BSI)**<br>V1 43, 52.6 ± 10.8<br>V2 43, 45.8 ± 8	CR<br>CR + CPI	53.5 + 12.6<br>51.6 + 8.7	18<br>19	44.5 + 7.2<br>47.1 + 8.7	15<br>17
**Compulsion (BSI)**<br>V1 43, 55 ± 10.1<br>V2 43, 48.1 ± 11.8	CR<br>CR + CPI	55.5 + 9.6<br>54.6 + 10.9	18<br>19	48 + 12.8<br>48.2 + 11	15<br>17
**BODI 1**<br>V1 37, 31.4 ± 18<br>V2 32, 27.4 ± 18.3	CR<br>CR + CPI	35.6 + 17.9<br>27.4 + 17.6	18<br>19	29.4 + 16.4<br>25.6 + 20.1	15<br>17
**BODI 2**<br>V1 37, 58.3 ± 31.3<br>V2 32, 57.5 ±31.7	CR<br>CR + CPI	59.3 + 28.9<br>57.3 + 34.2	18<br>19	57 + 31.8<br>57.8 + 32.5	15<br>17
**BODI 3**<br>V1 37, 34.8 ± 23<br>V2 32, 24.2 ± 17	CR<br>CR + CPI	30.3 + 18<br>39.2 + 26.6	18<br>19	22.5 + 16.5<br>25.7 + 17.7	15<br>17
**BODI 4**<br>V1 37, 45.2 ± 39.5<br>V2 32, 50 ± 37.4	CR<br>CR + CPI	45.2 + 40.6<br>45.2 + 39.5	18<br>19	50.1 + 41.6<br>49.9 + 34.5	15<br>17

Education Status n (%)	Level	CR	CR + CPI
	Secondary, 1st level	2 (4.7)	0
	Secondary, 2nd level	2 (4.7)	3 (7)
	Post-Secondary (non-tertiary)	9 (20.9)	7 (16.3)
	Post-Secondary (university)	9 (20.9)	11 (25.6)
**Occupation Status** n (%)	Self-Employed	10 (23.3)	8 (18.6)
	Employed	7 (16.3)	6 (14)
	Unemployed/Training/Household	0	3 (7)
	Retired/Pensioned	5 (11.6)	4 (9.3)

Supplementary Table S1. Sample characteristics for all variables with means, standard deviations and number of data entries, respectively for visit 1 (V1, baseline) and visit 2 (V2, day 15). Characteristics are provided for the total sample as well as the two groups defined by either caloric restriction (CR) alone or with add-on clinical psychological intervention (CPI).
